# The Obesity Paradox in Cancer: Is Bigger Better?

**DOI:** 10.1002/jcsm.13007

**Published:** 2022-05-04

**Authors:** Barry J.A. Laird, Richard J.E. Skipworth

**Affiliations:** ^1^ Institute of Genetics and Cancer University of Edinburgh Edinburgh UK; ^2^ St Columba's Hospice Care Edinburgh UK; ^3^ Caledonian Cachexia Collaborative Edinburgh UK; ^4^ Clinical Surgery University of Edinburgh, Royal Infirmary of Edinburgh Edinburgh UK

First described over 20 years ago in cardio‐metabolic disease, the obesity paradox is a medical hypothesis that being overweight can confer some form of overall survival advantage in a wide variety of illnesses. A body mass index (BMI) of 22.5 kg/m^2^ is the midpoint for normal weight. As BMI increases from the midpoint towards the level of being overweight (>25 kg/m^2^), it has been widely reported that this can be advantageous for patients, particularly in terms of survival.^1^

Multiple cancer studies have observed that being overweight has a survival advantage; however, this has not been uniformly reported in either individual studies or systematic reviews.^2^ Lennon and co‐workers highlighted that some of the challenges in dissecting findings include the BMI as an obesity measure as well as biases including detection, collider and reverse causality. They argue that further work is needed before the belief that ‘being obese might be good or protective for cancer patients’ is accepted. Caution is indeed advised.

In cancer, where previous large biorepository studies have demonstrated that low BMI and increased weight loss are associated with reduced survival, the concept of an obesity paradox would seem out of place.^3^ Yet, as our understanding of body composition and its role in determining clinical outcomes becomes increasingly understood, it lends itself to a deeper exploration of the obesity paradox. Lung cancer would seem a good place to start as it remains widely regarded as one of the most ‘cachectic cancers’, and is the leading cause of cancer death worldwide with sub‐optimal survival. Kichenadasse and co‐workers undertook a pooled analysis of four international clinical trials (*n* = 2110) examining the relationship between BMI and survival in patients with Non‐small cell lung cancer (NSCLC) being treated with immune checkpoint inhibitors.^4^ They observed that high BMI (>30 kg/m^2^) was associated with improved overall survival and proposed it should be considered as a stratification factor in future clinical trials. This analysis lacked granularity, however, in terms of body composition. Specifically, it was argued that future work should refine BMI into specifics of body composition, including lean and fat mass, to help understand the underlying biology.^5^, ^6^

The body of work led by Caan on body composition has sought to seek a deeper understanding of the true nature of the obesity paradox, arguing that the term ‘BMI paradox’ may be more appropriate; specifically, that low levels of lean mass are related to reduced survival and that people who are overweight have more muscle.^7^ Conversely, increases in visceral and total adiposity may be associated with worsened survival.^8^ It has been suggested that the survival ‘sweet spot’ for BMI may be in the range of 25–30 kg/m^2^ and that extremes to either side of this range are detrimental.^9^

Lee and co‐workers' have taken this work forward, and their examination of the specifics of body composition (including muscle) in relation to overall survival in NSCLC is published in this issue.^10^ They undertook a retrospective analysis of patients treated with surgical resection for NSCLC (*n* = 636) over a 5 year period assessing body composition using positron emission tomography‐computerized tomography (PET‐CT). Specifically, they examined skeletal muscle mass status, skeletal muscle index, and skeletal muscle area, as well as adipose tissue parameters. They observed that patients who were underweight had reduced survival as opposed to patients who were overweight and had increased survival. Of note, these associations were independent of other factors (e.g. tumour stage and co‐morbidities), and critically the ‘prognostic significance of obesity was independent of skeletal muscle’.

As the excellent study herein adds further weight to the obesity paradox, just as the authors acknowledge, we must be mindful of confounding factors. Banack and Stokes highlight that ‘paradoxes should be met with scepticism and counterintuitive results discussed with colleagues and collaborators’.^11^ The authors suggest that work testing their findings in other lung cancer stages (non‐surgical cohorts) and types, and the influence of smoking needs done. Indeed, it may be that the obesity paradox is tumour‐specific, and therefore extension of these sorts of studies to other tumour types is also required.^9^ Equally, it should be borne in mind that Lee and co‐workers have analysed a surgical cohort, and thus when considering the role of BMI in overall survival, one is also simultaneously considering the role of BMI in post‐operative recovery, disease recurrence and non‐cancer death. The relative impact of BMI on the latter two determinants of survival is currently poorly understood, but it has been shown that visceral adiposity is associated with increased cardiovascular risk in breast cancer survivors, and cardiovascular events (including myocardial infarction, stroke and cardiovascular death) in colorectal cancer.^12^

We remain unclear as to the true nature of the obesity paradox and whether or not simply being overweight has a survival advantage in certain cancers. However, as our understanding of cancer cachexia improves and its acknowledgement as primarily a metabolic rather than nutritional syndrome is increasingly accepted, other factors including the systemic inflammatory response need to be explored as potential confounding factors.^13^ To illustrate, Martin et al demonstrated that weight loss is determined by dietary intake and systemic inflammation^14^ and that functional status combined with inflammatory status are the main determinants of quality of life.^15^, ^16^ Conversely, the value of lean mass in survival prediction has also been questioned in that values appear similar across multiple cancer types of differing stages.^17^

As a research community, we must continue to disentangle the complex mechanisms in our midst through data‐sharing and interrogation of findings where logic is questioned. We hope the paper herein provides a catalyst for further work on the obesity paradox in cancer.

## Conflict of interest

The authors declared no conflict of interest.
